# The prospective application of a hypoxic radiosensitizer, doranidazole to rat intracranial glioblastoma with blood brain barrier disruption

**DOI:** 10.1186/1471-2407-13-106

**Published:** 2013-03-08

**Authors:** Hironobu Yasui, Taketoshi Asanuma, Junichi Kino, Tohru Yamamori, Shunsuke Meike, Masaki Nagane, Nobuo Kubota, Mikinori Kuwabara, Osamu Inanami

**Affiliations:** 1 Laboratory of Radiation Biology, Department of Environmental Veterinary Sciences, Graduate School of Veterinary Medicine, Hokkaido University, Kita 18 Nishi 9, Kita-ku, Sapporo, Hokkaido, Japan; 2 Laboratory of Veterinary Radiology, Department of Veterinary Sciences, University of Miyazaki, 1-1, Gakuen Kibanadai-nishi, Miyazaki, Miyazaki, Japan; 3 POLA PHARMA INC, 8-9-5, Nishigotanda, Shinagawa-ku, Tokyo, Japan

**Keywords:** Doranidazole, Radiosensitizer, Glioblastoma, Hypoxia

## Abstract

**Background:**

Glioblastoma is one of the intractable cancers and is highly resistant to ionizing radiation. This radioresistance is partly due to the presence of a hypoxic region which is widely found in advanced malignant gliomas. In the present study, we evaluated the effectiveness of the hypoxic cell sensitizer doranidazole (PR-350) using the C6 rat glioblastoma model, focusing on the status of blood brain barrier (BBB).

**Methods:**

Reproductive cell death in the rat C6 glioma cell line was determined by means of clonogenic assay. An intracranial C6 glioma model was established for the *in vivo* experiments. To investigate the status of the BBB in C6 glioma bearing brain, we performed the Evans blue extravasation test. Autoradiography with [^14^C]-doranidazole was performed to examine the distribution of doranidazole in the glioma tumor. T2-weighted MRI was employed to examine the effects of X-irradiation and/or doranidazole on tumor growth.

**Results:**

Doranidazole significantly enhanced radiation-induced reproductive cell death *in vitro* under hypoxia, but not under normoxia. The BBB in C6-bearing brain was completely disrupted and [^14^C]-doranidazole specifically penetrated the tumor regions. Combined treatment with X-irradiation and doranidazole significantly inhibited the growth of C6 gliomas.

**Conclusions:**

Our results revealed that BBB disruption in glioma enables BBB-impermeable radiosensitizers to penetrate and distribute in the target region. This study is the first to propose that in malignant glioma the administration of hydrophilic hypoxic radiosensitizers could be a potent strategy for improving the clinical outcome of radiotherapy without side effects.

## Background

Glioblastoma, a highly malignant brain tumor, usually has a poor prognosis despite surgical treatment, radiation therapy and/or chemotherapy [1,2]. Even when recognizable tumor mass can be surgically removed and adjuvant radiotherapy and chemotherapy are employed, the mean survival of patients is only extended from 2–3 months to 1 year [3]. Several factors are considered to be responsible for the radioresistance of glioblastomas such as hypoxia [4], the up-regulation of the EGFR pathway [5] and the existence of glioma stem cells [6]. Tumor hypoxia, which is generally attributed to the imbalance between the demand and supply of oxygen and poorly organized vasculature [7,8], is observed in many tumor types especially glioblastoma. Hypoxia appears to be the most important factor in the development of radioresistance, invasiveness and more aggressive tumor phenotypes [9]. Therefore, to develop therapies against glioblastoma, an invariably fatal disease, enhancement of the efficacy of radiotherapy by means of hypoxic radiosensitizers is certainly a promising way to achieve improved therapeutic outcome.

Numerous radiosensitizers for hypoxic cells have been developed and screened, both in preclinical studies and clinical trials [10,11]. The nitroimidazole derivatives are major compounds in this regard and have been tested extensively. However, most clinical trials have failed to demonstrate significant efficacy using these sensitizers, mainly because of undesirable side effects such as neurotoxicity [12]. However, clinical trials in Denmark reported that misonidazole and nimorazole were effective in chemoradiotherapy against carcinomas of the larynx and pharynx [13,14]. The efficacy of nitroimidazole derivatives as hypoxic radiosensitizers remains controversial. It is currently difficult to determine which type of tumor is susceptible to hypoxic radiosensitization and which regimen is most efficient using nonproprietary drugs, because of the lack of financial incentives for the pharmaceutical industries to evaluate them [11].

Doranidazole (1-[1’,3’,4’-trihydroxy-2’-butoxy]-methyl-2-nitroimidazole [PR-350]) is a hypoxic radiosensitizer, and is a derivative of 2-nitroimidazole intended to reduce neurotoxicity due to its blood brain barrier (BBB) impermeability [15,16]. Several studies have shown that doranidazole has a radiosensitizing effect under hypoxia, both *in vitro*[17-19] and *in vivo*[19-21]. Based on these studies, a phase III trial of doranidazole against advanced pancreatic cancer was performed; it was demonstrated that treatment with doranidazole following radiation significantly improved the tumor mass reduction rate and extended patient survival [22]. While various results have suggested that doranidazole has promising potential in hypoxia-targeting chemoradiotherapy, to date there have not been any reports on the use of this drug for intracranial glioma.

It is known that the BBB restricts the transport of hydrophilic or high-molecular-weight compounds into the brain to maintain the brain internal milieu. Therefore, doranidazole, which has a hydrophilic residue, cannot cross the BBB and cause any toxicity to the intact brain. However, in many advanced malignant gliomas, disruption of the BBB has been reported [23-25]. These facts led us to consider the possibility that doranidazole might only reach the tumor regions and not the surrounding healthy brain.

In the present study, we examined the radiosensitizing effect of doranidazole on C6 glioma both *in vitro* and *in vivo*. We particularly focused on the extent of BBB disruption in C6-bearing rat brain and also investigated the uptake of doranidazole in the tumor region.

## Methods

### Materials

Doranidazole and 2’-[^14^C]-labeled doranidazole ([^14^C]-doranidazole) were supplied by POLA PHARMA INC. (Tokyo, Japan). The Hypoxyprobe™-1 Kit was obtained from Hypoxyprobe Inc. (Burlington, MA, USA). A BD Matrigel™ reagent was purchased from BD Biosciences (Billerica, MA, USA). Ultrapure N_2_ gas (99.999%) was obtained from Air Water Technical Supply (Ishikari, Japan). Other chemicals were purchased from Wako Pure Chemical Industries, Ltd. (Tokyo, Japan) unless otherwise stated.

### Cell culture

Rat glioma cell line C6 was obtained from the Health Science Research Resources Bank (Osaka, Japan). The cells were maintained in Dulbecco’s modified Eagle’s medium (DMEM; Gibco-BRL/Invitrogen, Carlsbad, CA, USA) supplemented with 10% fetal bovine serum (FBS: Filtron, Brooklyn, Australia) at 37°C in 5% CO_2_/95% air.

### Cell incubation, X-irradiation and drug treatment *in vitro*

Tumor cells attached to a 6-cm plastic dish were treated with 10 mM doranidazole before hypoxic incubation. The hypoxic condition (oxygen concentration ≤ 10 mmHg [1.3%]; unpublished data) for tumor cells in the dish was achieved by placing it in a gas-exchangeable chamber [18] and continuously passing ultrapure N_2_ gas for 25 minutes on ice. The cells were then exposed to 20 Gy of X-rays while maintaining the gas flow. X-irradiation was performed with a Shimadzu PANTAK HF-350 X-ray generator (1.0 mm Al filter; 200 kVp; 20 mA; Shimadzu, Kyoto, Japan).

### Clonogenic survival assay

After X-irradiation under hypoxia or normoxia, C6 cells were collected by trypsinization and washed with PBS. The proper number (200–30000) of cells were seeded on a 6-cm plastic dish containing fresh medium with 10% fetal bovine serum, followed by incubation at 37°C for 8 days. The cells were then fixed with methanol, stained with Giemsa solution and scored under a microscope. Only colonies containing more than 50 cells were scored as surviving cells. The surviving fraction at each dose was calculated with respect to the plating efficiency of the nonirradiated control.

### Animals

WKAH/Hkm rats aged 9 weeks were purchased from Japan SLC (Hamamatsu, Japan). All animal experiments in this study were conducted according to the guidelines of the Law for The Care and Welfare of Animals in Japan and approved by the Animal Experiment Committee of the Graduate School of Veterinary Medicine, Hokkaido University.

### Intracranial tumor model

The C6 intracranial tumor model was established according to the method detailed in our previous study [26]. Anesthetized rats were placed on a stereotaxic device (Narishige Scientific Instrument Lab., Tokyo, Japan). A 1-mm hole was drilled through the skull 2 mm anterior and 2 mm lateral to the bregma on the right-hand side of the head. One million of C6 cells in a mixture of 5 μL FBS(−) culture media and 5 μL Matrigel were injected into the cortex at a 3-mm depth at a rate of 2 μL/min. A waiting time of 2 minutes was implemented following injection and the hole was closed using bone wax. The incision was sutured and covered with surgical glue.

### Evaluation of the BBB disruption in C6-bearing rats

Vascular permeability in C6-bearing brain was evaluated by perfusing it with Evans blue dye according to the method described previously [27]. In brief, Evans blue dye solution (2%) was intravenously administered to rats at a dose of 3 ml/kg and allowed to circulate for 60 minutes. To remove intravascular dye, rats were transcardially perfused with saline for 20 minutes. Brains were removed and sectioned at a thickness of 2 mm.

### Treatment with doranidazole and X-irradiation

Doranidazole administration and X-irradiation were performed when the tumor reached a size of 50–100 mm^3^. Animals were randomized into four groups: (1) no treatment; (2) X-irradiation (6 Gy) alone; (3) doranidazole administration alone; and (4) doranidazole administration at 30 minutes before X-irradiation (6 Gy). Doranidazole at a dose of 200 mg/kg was intravenously (i.v.) injected into rats. For irradiation of intracranial tumors, rats were shielded with lead panels, except for the tumor-bearing cranium. X-irradiation was performed with a Shimadzu PANTAK HF-350 X-ray generator at a dose rate of 1.2 Gy/min.

### MRI experiments

MRI was carried out using a 7.05 T superconducting magnet (Oxford Instruments, Oxford, UK) equipped with a Unity/Inova 300/183 spectrometer (Varian, Palo Alto, CA, USA). Rats were placed in the center of a 35 mm diameter quadrature RF coil. After rapid assessment of the tumor position using a multislice spin-echo (MSE) sequence, T2-weighted images (T2WIs) were also obtained using a MSE sequence with TR/TE = 2000 ms/60 ms, FOV = 80 × 80 and 60 × 60 mm (for sagittal and coronal images, respectively), image matrix = 128 × 128 and slice thickness = 1 mm. Using lengths of tumors measured in three orthogonal dimensions, tumor volume (*V*) was calculated as: *V* (mm^3^) = π(*a* × *b* × *c*)/6, where *a, b* and *c* represent width, height and thickness, respectively.

To measure leakage from the BBB, a gadolinium-chelate (Gd-[DTPA]) contrast material (Magnevist®, gadopentetate dimeglumine: Bayer Healthcare Pharmaceuticals, Montville, NJ, USA) was i.v. injected at a concentration of 0.1 mmol/kg body weight. Contrast-enhanced MRI (CE-MRI) images were obtained using multislice T1-weighted images (T1WIs) with spin-echo sequences. The parameters of the CE-MRI were TR/TE = 500 ms/16 ms, slice thickness = 1 mm, FOV = 51.2 × 51.2 mm, and image matrix = 256 × 256. The quantification of the signal enhancement due to Gd-[DTPA] uptake to glioma was performed using Image J software (National Institutes of Health, Bethesda, MD, USA) by calculating the ratio of signal intensity in tumor region to that in normal brain region.

### Autoradiography

To examine the distribution of doranidazole in the rat brain, we performed autoradiographic analysis using [^14^C]-doranidazole. Tumor-bearing rats were i.v. injected with 500 μL of [^14^C]-doranidazole (4.9 MBq/head). At 90 minutes after drug administration, rats were decapitated without prior perfusion with saline. Their brains were immediately removed and frozen. Frozen sections that were 20-μm thick were exposed to a radiosensitive imaging plate (BAS-SR2040: Fuji Film Co. Ltd., Tokyo, Japan) for 4 days with a radioactive standard slide (ARC-146: American Radiolabeled Chemicals Inc., St Louis, MO, USA). The image acquisition was performed using a BAS-2500 Bioimage Analyzer system (Fuji Film Co. Ltd. Tokyo, Japan). After the acquisition of autoradiographic images, parts of sections were fixed with 4% buffered formaldehyde and stained with hematoxylin/eosin (H/E).

### Immunohistochemistry

At 1 day after treatment with doranidazole and/or X-irradiation tumor-bearing rats were i.v. injected with pimonidazole (Hypoxyprobe™-1 Kit; 60 mg/kg). At 90 minutes after drug administration, rats were perfused with saline and subsequently 4% buffered formaldehyde. Removed brain tissues were fixed, embedded in paraffin and sectioned at 5-μm thickness. The immunostaining procedure for pimonidazole was carried out in accordance with the manufacturer’s instructions. Serial sections were also stained with H/E. The stained images of each section were acquired using a fluorescence microscope (BZ-9000: Keyence, Osaka, Japan).

### Statistical analysis

All results were expressed as the mean ± S.E. The variance ratio was estimated using the F-test and differences in means of groups were determined using Student’s t-test or Welch’s t-test. The minimum level of significance was set at P < 0.05.

## Results

**Figure 1:**
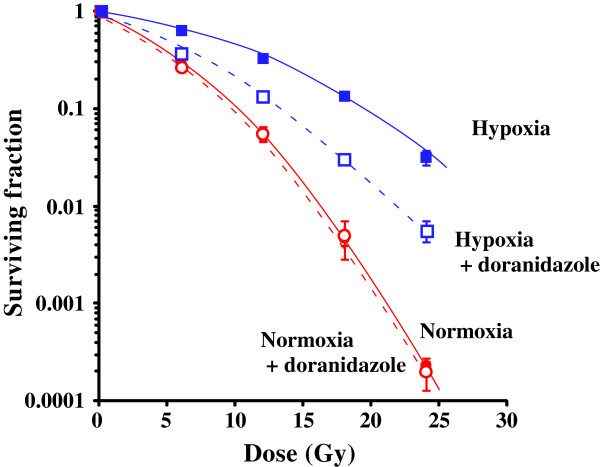
**Sensitization of C6 cells to radiation under hypoxia using doranidazole.** Dose–response curves of X-irradiated C6 cells. Tumor cells were X-irradiated under normoxia (*red closed circles*), under normoxia with doranidazole (*red open circles*), under hypoxia (*blue closed squares*) and under hypoxia with doranidazole (*blue open squares*). The surviving fraction at each dose was calculated and corrected according to the plating efficiency of the nonirradiated control. Data are expressed as the mean ± S.E. for three experiments.

The clonogenic survival curves for C6 glioma cells irradiated *in vitro* under normoxic and hypoxic conditions, with or without doranidazole, are shown in Figure 1. Under conditions without doranidazole, X-irradiation under hypoxia reduced the radiosensitivity of C6 cells, and the oxygen enhancement ratio (OER) was approximately 1.9. The hypoxic condition set in this experiment was ≤ 10 mmHg for pO_2_, and this OER value coincided with that reported in a previous study [28]. Under normoxic conditions without irradiation, the survival fractions with or without doranidazole were 0.703 ± 0.019 and 0.677 ± 0.031, respectively. Hypoxic conditions decreased the plating efficiency of C6 cells to 0.675 ± 0.006 and the addition of doranidazole resulted in a further decline to 0.667 ± 0.032, although no significant differences were observed among the groups. Under both normoxia and hypoxia without irradiation, the toxicity of 10 mM doranidazole against C6 cells was less than 30%. While doranidazole had no sensitizing effect when combined with aerobic irradiation, it had significant sensitizing activity when combined with irradiation under hypoxic conditions. The dose that reduces cell survival to 10% (D_10_) obtained from the hypoxic cell survival curve was 20.2 Gy, and it decreased to 13.3 Gy when cells were irradiated in the presence of 10 mM doranidazole. The sensitizing enhancement ratio (SER) for doranidazole after irradiation under hypoxic conditions was ~1.5, whereas the SER after irradiation under normoxic conditions was ~1.0.

**Figure 2:**
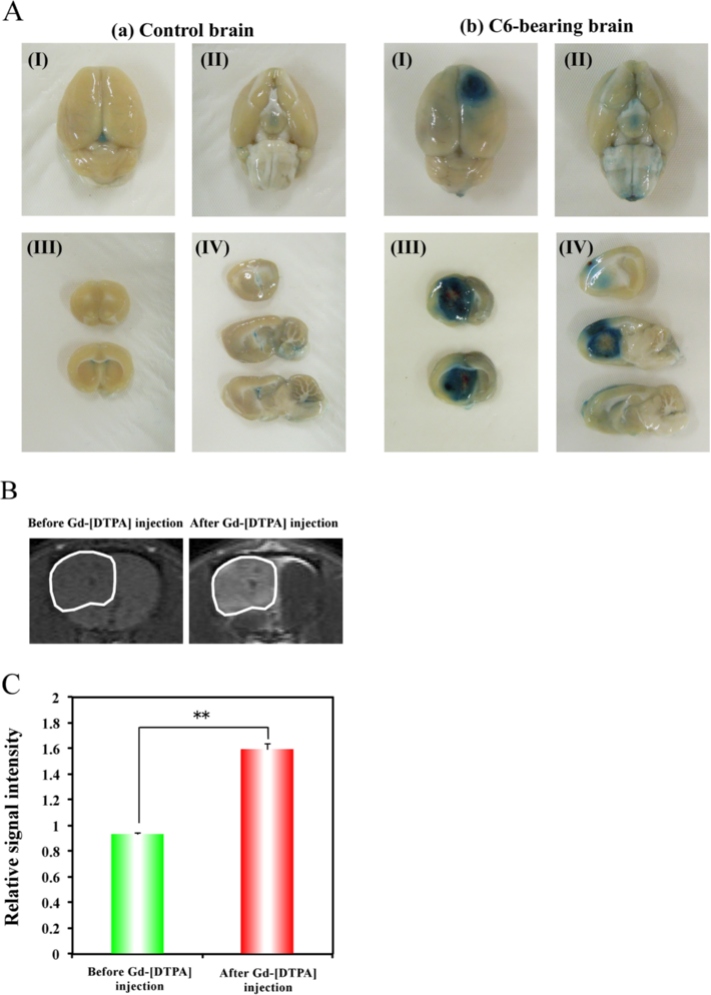
**Disruption of the BBB in the brain of a C6-bearing rat.** (**A**) Representative photographs of the dorsal surface (**I**), ventral surface (**II**), coronal slice (**III**) and sagittal slice (**IV**) of control brain (a) and C6-bearing brain (b) after perfusion with Evans blue dye. (**B**) Representative T1-weighted MR images obtained before and after Gd-[DTPA] injection. White lines show the region with high signal intensity, indicating the BBB-disrupted region. (**C**) Quantitative data for Gd-[DTPA]-based CE-MRI. Relative MRI signal intensities are expressed as ratios relative to the normal brain region.

To examine the disruption of the BBB in the C6-tumor-bearing rat brain, we employed the Evans blue extravasation method. Evans blue dye is known to bind to albumin producing a 68 kDa compound that does not cross the BBB [29]. In fact, normal control brain after intra-arterial infusion of Evans blue showed no staining in the cerebral hemisphere (Figure 2A [a-I, II]). Using this Evans blue extravasation test, we evaluated the permeability of the BBB in C6-bearing brain. Figure 2A (b-I, II) shows a clearly stained region in the frontal cortex of right hemisphere, in which the C6 tumor was located. The photographs in Figure 2A (III, IV) are views of sectioned slices from control and C6-bearing brains. They also demonstrated the apparent correspondence of the stained region with the tumor region in C6-bearing brain, while no staining was observed in the control brain. To confirm this disruption of the BBB in the tumor region, we performed CE-MRI analysis using a BBB-impermeable reagent, Gd-[DTPA]. Figure 2B displays representative pre- and post-contrast T1WIs of brains in C6-glioma-bearing rats, with the region of interest (ROI) placed on the glioma. After Gd-[DTPA] injection, MRI signal enhancement due to the accumulation of Gd-[DTPA] was clearly observed around the tumor region. The quantitative data showed that the relative signal intensities in glioma before and after Gd-[DTPA] injection were 0.933 ± 0.008 and 1.597 ± 0.042, respectively (Figure 2C).

**Figure 3:**
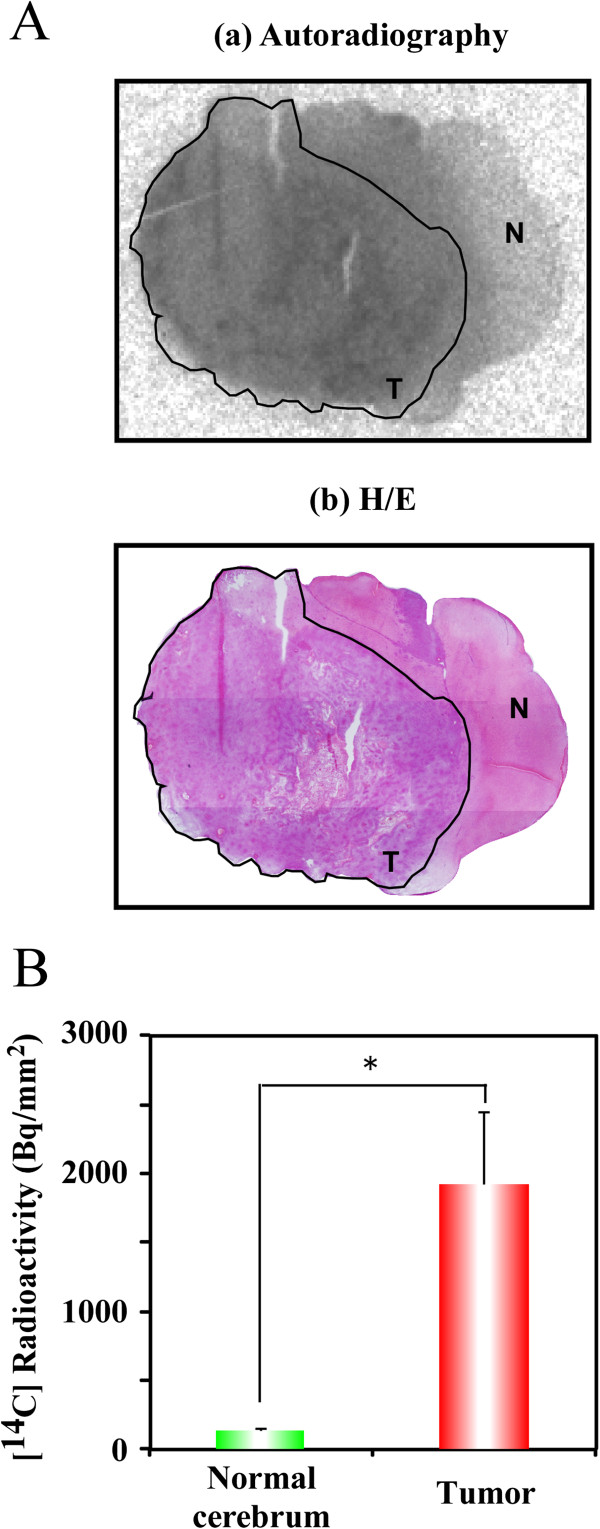
**The distribution of [**^**14**^**C]-doranidazole in C6 intracranial glioma.** (**A**) A 20-μm thick tissue section of rat brain that was used for autoradiography (a) and subsequent H/E staining (b). Black lines show the C6 glioma. The annotated words “T” and “N” represent tumor and normal brain regions, respectively. (**B**) Using these images, quantitative data for the accumulation of [^14^C]-doranidazole in normal cortex and C6 glioma was acquired. Data are expressed as the mean ± S.E. for four different tumors. *: P < 0.05 *vs.* normal cerebrum.

We next investigated the distribution of doranidazole in the brains of C6-bearing rats. Ninety minutes after the i.v. administration of [^14^C]-doranidazole, rats were decapitated. Brain tissue sections were analyzed using autoradiography and subsequent H/E staining. In the autoradiographic image shown in Figure 3A(a), [^14^C]-doranidazole is clearly distributed in the tumor region but not in the normal brain cortex. We then quantified the accumulation of [^14^C]-doranidazole in each region of the normal cortex and tumor region defined by H/E staining (Figure 3A[b]). Tumor regions showed significantly higher [^14^C] radioactivity levels (1926.5 ± 523.3 Bq/mm^2^) than the normal cortex region (138.7 ± 14.6 Bq/mm^2^) (Figure 3B). These results suggested that doranidazole could penetrate into the tumor region due to the breakdown of the BBB in the C6-bearing brain.

**Figure 4:**
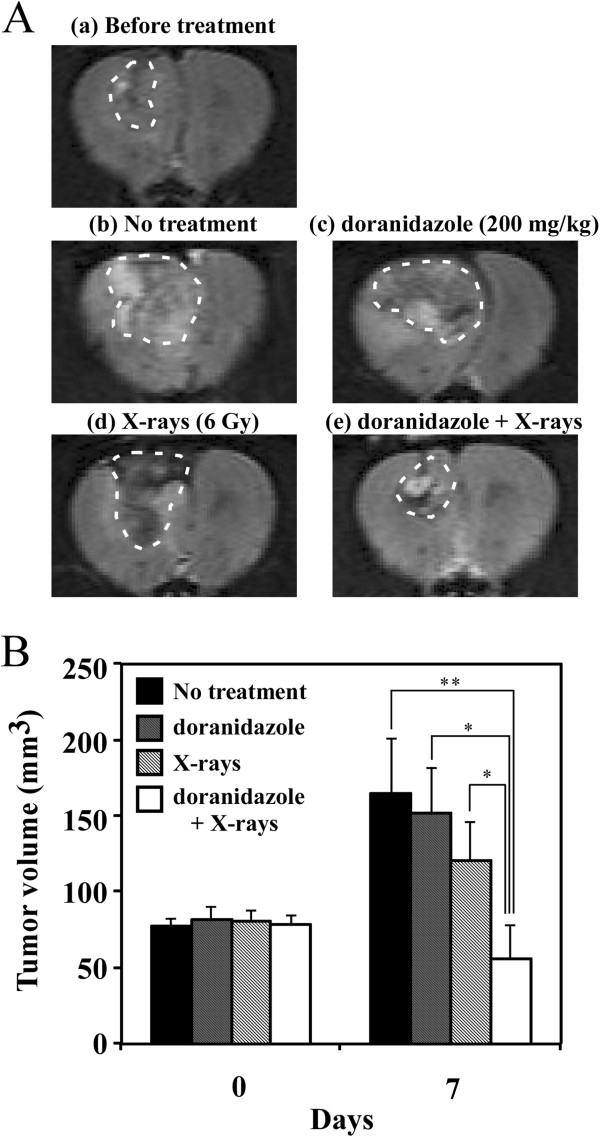
**Effects of the combination of doranidazole and X-irradiation on tumor growth in C6 glioma.** When the tumor reached a size of 50–100 mm^3^, rats were treated with doranidazole (200 mg/kg) and/or X-irradiation (6 Gy). (**A**) Typical T2-weighted MR images of a C6-bearing brain before and after each treatment. (**B**) The quantitative data for suppression of tumor growth by doranidazole administration and/or X-irradiation. The sizes of tumors were estimated using T2-weighted MRI before treatment and at 7 days after treatment. Data are expressed as the mean ± S.E. for 5–8 different tumors. *: P < 0.05, **: P < 0.01.

**Figure 5:**
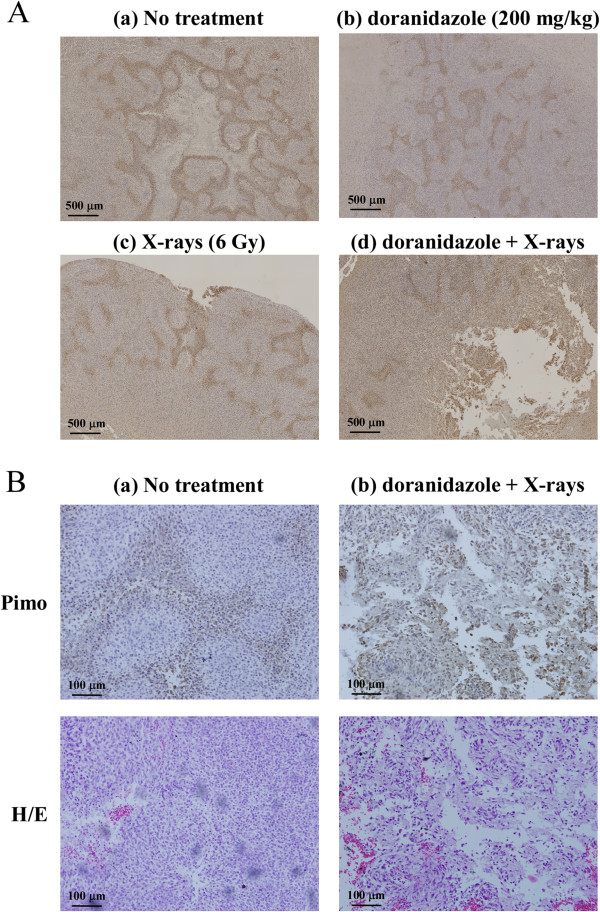
**Effects of the combination of doranidazole and X-irradiation on tumor hypoxia in C6 glioma.** Histological evaluation of C6 tumors at 1 day after treatment. (**A**) Immunohistochemical images for pimonidazole. Animals received vehicle (a), doranidazole (200 mg/kg) (b), 6 Gy of X-rays (c), or a combination (d) as described in Figure 4. A representative field for each condition is shown. Bar = 500 μm. (**B**) Representative images of pimonidazole staining and H/E staining taken at high magnification in C6 tumors resected from the control group (a) and the combination group (**b**). Bar = 100 μm.

We also examined the radiosensitizing effect of doranidazole on the growth of transplanted C6 glioma. Rats with 50–100 mm^3^ of glioma tumor were treated with 200 mg/kg doranidazole and/or 6 Gy of X-rays. We estimated the tumor volumes before and after each treatment using T2WIs to indicate the definite tumor area (Figure 4A). As shown in Figure 4B, without any treatment tumor size increased ~2.5-fold in 7 days and reached 165.3 ± 35.5 mm^3^. X-irradiation or doranidazole alone induced no statistically significant inhibition of tumor growth. The tumor volumes at 7 days after treatment were 121.0 ± 24.9 mm^3^ after X-irradiation alone and 152.0 ± 30.3 mm^3^ after doranidazole alone. X-irradiation at 30 minutes after doranidazole treatment induced a significant retardation in tumor growth (56.0 ± 22.7 mm^3^). To examine the suppressive effect of doranidazole on the hypoxic region in the C6 glioma, histological analysis with pimonidazole staining and H/E staining was performed. Immunohistological images for pimonidazole revealed a characteristic cord-like structure of hypoxia in viable tumor, within specimens resected from tumors receiving radiation or doranidazole alone. However, the great majority of the tumor containing hypoxic region was necrotic after combined treatment (Figure 5).

## Discussion

In the present study, we investigated the radiosensitizing effect of a hypoxic cell radiosensitizer, doranidazole, on C6 intracranial glioma. Doranidazole has a 2-nitroimidazole-based chemical structure with a side chain having low lipophilicity. It is designed to be less neurotoxic due to its BBB-impermeability [15,16]. In common with other 2-nitroimidazole derivatives such as misonidazole and etanidazole, doranidazole is reduced under hypoxic conditions and imported into the cell nucleus, leading to fixation of radiation damage in a manner similar to oxygen [30]. In the present study, it was clearly demonstrated *in vitro* that doranidazole radiosensitized hypoxic cells as determined by clonogenic survival assay (Figure 1). This radiosensitizing effect was consistent with previous reports [15,21].

Because the delivery of hydrophilic doranidazole into the tumor region is crucial for its radiosensitizing effect, we investigated the extent of the BBB disruption using Evans blue dye extravasation. Figure 2A clearly shows the penetration of this dye into the tumor region, but not normal brain tissue. The disrupted BBB allows MR-based detection of glioblastoma by extravasation and accumulation of contrast agents such as Gd-DTPA in the interstitial spaces [31]. By using this method, the breakdown of the BBB in C6 glioma was confirmed by CE-MRI with Gd-[DTPA] (Figure 2B and C). Due to its trihydroxyl structure, doranidazole is less lipophilic than misonidazole and etanidazole, with reduced neurotoxicity. The disruption of the BBB as shown in Figure 2 may indicate the feasibility of using doranidazole to treat some intracranial tumors. In fact in the current study, autographic analysis *in vivo* indicated the obvious accumulation of [^14^C]-doranidazole in the tumor region. To our knowledge, our results have clarified for the first time that disruption of the BBB, which has been observed in some types of glioblastoma such as C6 glioma, enabled a lipophobic nitroimidazole analog, doranidazole to be incorporated into the tumor region. To reveal the variability in tumor response to doranidazole based on levels of hypoxia, further investigation using other glioma models will be required.

As mentioned, a number of clinical trials involving a few 2-nitroimidazole-derivatives in combination with radiotherapy have been performed with the objective of improving therapeutic benefit. However, most of them have provided disappointing results with poor enhancement of the efficacy of radiotherapy and severe side effects such as neurotoxicity. To develop an effective therapy with few side effects and sufficient radiosensitizing effects, it is necessary to identify the appropriate tumor type using optimal parameters such as oxygenation status and vascular permeability. Currently, several noninvasive tools are being established for the monitoring of tumor oxygenation and blood perfusion [32,33]. To confirm the rationale for using hypoxic cell sensitizers, microenvironmental information on the target tumor should be obtained in preclinical and clinical studies.

## Conclusions

In conclusion, we demonstrated that doranidazole had a radiosensitizing effect on C6 glioma, a tumor model that shows a wide range of hypoxia and disruption of the BBB. The observation of synergistic tumor growth inhibition by combined treatment with X-irradiation and doranidazole, as shown in Figure 4, clearly indicates the possibility of clinical administration of this drug in the treatment of intracranial glioma. Our study also demonstrated that this radiosensitization effect was induced through the selective accumulation of doranidazole in a BBB-disrupted tumor. Thus, doranidazole may be a candidate radiosensitizer for use against malignant glioma.

## Abbreviations

BBB: Blood brain barrier; DMEM: Dulbecco’s modified Eagle’s medium; FBS: Fetal bovine serum; i.v.: intravenous; MSE: Multislice spin-echo; T2WI: T2-weighted image; Gd-DTPA: Gadopentetate dimeglumine; CE-MRI: Contrast-enhanced MRI; T1WI: T1-weighted image; H/E: Hematoxylin/eosin; SER: Sensitizing enhancement ratio; ROI: Region of interest.

## Competing interests

NK is an employee of POLA PHARMA INC.; all of the other authors have no competing interests to declare.

## Authors’ contributions

HY, TA and JK performed the *in vitro* and *in vivo* experiments, analyzed the data and prepared the manuscript. TY and SM also participated in the performance of the *in vitro* experiments. MN prepared the glioma-transplanted animal model. NK synthesized doranidazole and [^14^C]-doranidazole. MK and OI designed the research and interpreted the data. All authors approved the final version of the manuscript.

## Pre-publication history

The pre-publication history for this paper can be accessed here:

http://www.biomedcentral.com/1471-2407/13/106/prepub
